# Rheology of Suspensions Thickened by Cellulose Nanocrystals

**DOI:** 10.3390/nano14131122

**Published:** 2024-06-29

**Authors:** Rajinder Pal, Karthika Pattath

**Affiliations:** Department of Chemical Engineering, University of Waterloo, Waterloo, ON N2L 3G1, Canada; karthika.pattath@uwaterloo.ca

**Keywords:** rheology, non-Newtonian, shear-thinning, viscosity, suspension, nanocrystals, cellulose nanocrystals, nanoparticles, power-law model

## Abstract

The steady rheological behavior of suspensions of solid particles thickened by cellulose nanocrystals is investigated. Two different types and sizes of particles are used in the preparation of suspensions, namely, TG hollow spheres of 69 µm in Sauter mean diameter and solospheres S-32 of 14 µm in Sauter mean diameter. The nanocrystal concentration varies from 0 to 3.5 wt% and the particle concentration varies from 0 to 57.2 vol%. The influence of salt (NaCl) concentration and pH on the rheology of suspensions is also investigated. The suspensions generally exhibit shear-thinning behavior. The degree of shear-thinning is stronger in suspensions of smaller size particles. The experimental viscosity data are adequately described by a power-law model. The variations in power-law parameters (consistency index and flow behavior index) under different conditions are determined and discussed in detail.

## 1. Introduction

Suspensions are dispersions of solid particles in liquids. They are very important industrially, as many products of commercial importance are sold or handled in the form of suspensions. Industries where suspensions are encountered include foods, pharmaceuticals, petroleum, ceramics, mineral processing, construction, cosmetics and toiletries, paints, agriculture, biotechnology, polymers, and many more [1,2,3,4,5,6,7,8,9,10,11]. A good understanding of the rheology of suspensions is essential in the formulation, mixing, processing, transport, and storage of suspensions. One problem with suspensions is that they are prone to phase separation under the influence of gravity. For example, the particles of the suspension would settle at the bottom of the container if the particles were heavier than the suspending medium liquid. As particles of suspension are almost always of different density than the suspending medium, the matrix phase is often thickened to minimize phase separation in suspensions. To that end, thickeners or rheology modifiers are incorporated into the matrix phase of suspensions. According to the Stokes law, the settling velocity of a particle is given as follows [12]:

(1) $$U_{t}=\frac{\left(\rho_{p}-\rho \right)gd_{p}^{2}}{18\mu }\;$$

where $U_{t}$ is the terminal settling velocity of a particle, $\rho_{p}$ is density of particle, g is acceleration due to gravity, $d_{p}$ is particle diameter, and ρ and μ are matrix fluid density and viscosity, respectively. Thus, sedimentation of particles can be minimized by increasing the matrix viscosity and reducing the particle diameter assuming that the density difference between the particle and the matrix fluid is fixed.

The thickeners used in the formulation of suspensions include polymers, clays, surfactants, and nanoparticles [13]. However, more recently, nanocrystalline cellulose (NCC) is receiving a lot of attention as a rheology modifier or thickener of matrix phase due to the high aspect ratio and surface charge of nanocrystals. Nanocrystalline cellulose, also referred to as cellulose nanocrystals, is a promising low-cost nanomaterial with many potential applications [14,15,16,17,18,19,20,21,22,23,24,25,26]. It is non-toxic, biodegradable, and renewable. It is producedfrom the most abundantly available biopolymer on our planet, namely, cellulose by sulfuric acid hydrolysis of amorphous portions of cellulose fibers. The nanocrystals of NCC are rod-shaped and possess a negative charge when dispersed in water. 

In this study, we report new results on the rheology of suspensions thickened by nanocrystalline cellulose covering broad ranges of NCC and particle concentrations. Two different-size particles were used. The NCC concentration varied from 0 to 3.5 wt% based on the matrix liquid. The particle volume fraction varied from 0 to 0.57. The effects of pH and salt concentration on the rheology of suspensions were also investigated. To our knowledge, this is the first study to investigate the rheology of suspensions thickened by cellulose nanocrystals. Previous work on the rheology of suspensions, for the most part, is restricted to suspensions of particles in homogeneous Newtonian liquids and to suspensions of particles in non-Newtonian polymeric liquids [4,6,8,10,11,13].

## 2. Materials and Methods

### 2.1. Materials

Two different types of particles with different particle sizes were used: Low-density hollow spherical particles (trade name Extendospheres TG hollow spheres) with a Sauter mean diameter of 69 μm and solid spherical particles (trade name solospheres S-32) with a Sauter mean diameter of 14 μm. The particles were supplied by Sphere One, Inc., Chattanooga, TN, USA. Extendospheres TG hollow particles are low-density, high-strength ceramic particles used extensively in sealants, roofing compounds, caulks, adhesives, and latex flooring. Solospheres S-32 are solid ceramic particles used in the manufacturing of anti-skid floor coatings, gaskets, cementitious coatings, insulative roof coatings, chemical resistance coatings, and naval cements. 

**Figure 1:**
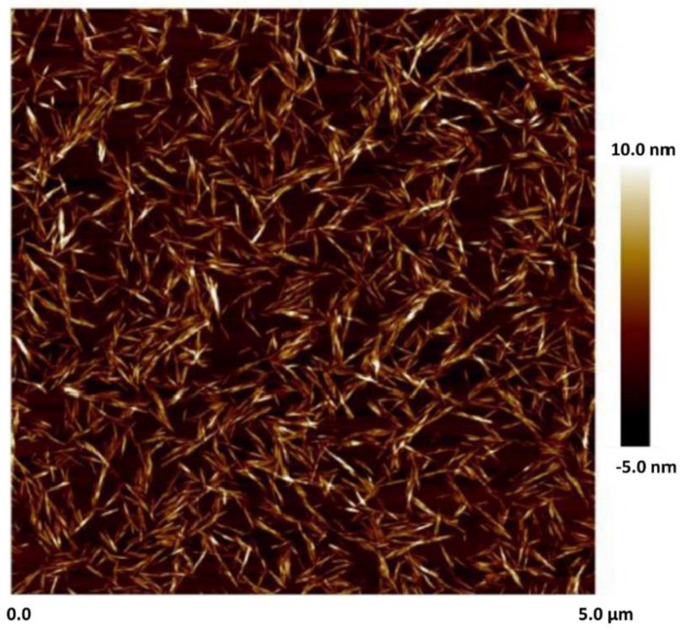
AFM image of NCC [27].

Cellulose nanocrystals, that is, NCC, was supplied by CelluForce Inc., Windsor, ON, Canada, under the trade name of NCC NCV100-NASD90. The nanocrystals were manufactured using sulfuric acid hydrolysis of wood pulp. Figure 1 shows the atomic force microscopy (AFM) image of cellulose nanocrystals. Clearly, the nanocrystals are rod-shaped particles. 

### 2.2. Preparation of Dispersions of Cellulose Nanocrystals

The dispersions of cellulose nanocrystals (NCC) were prepared at room temperature (≅23 °C) by slowly dispersing a known amount of NCC into a known amount of deionized water. The mixing of the dispersion was maintained using a turbine homogenizer (Gifford-Wood, model 1L) at a fixed speed. The dispersion was homogenized for at least 60 min for the nanocrystals to disperse and homogenize fully. Six differently concentrated NCC dispersions (0.25, 0.50, 1.0, 1.5, 2.5, and 3.5 wt%) were prepared. 

### 2.3. Preparation of Suspensions of Solid Particles in NCC Dispersion

Suspensions were prepared at room temperature (≅23 °C) by adding a known amount of particles (Extendospheres TG hollow spheres or solospheres S-32) to a known amount of NCC dispersion. Gentle mixing of the fluids was maintained during the addition of particles to the NCC dispersion using the homogenizer. The suspension was finally homogenized in the homogenizer at a high speed for at least 30 min after the addition of the particles to the NCC dispersion. To increase the particle concentration of the suspension, a known amount of more particles was added slowly to an existing lower particle concentration suspension and the mixture was homogenized at a high speed for at least 30 min.

### 2.4. Measurements

The size distribution of NCC was measured using dynamic light scattering (DLS) carried out in a Zetasizer Nano ZS90 manufactured by Malvern Instruments Ltd., Worcester, UK. The samples, consisting of a very dilute dispersion of NCC in water, were tested in ZEN0112, a low-volume disposable sizing cuvette, at 25 °C. A 120 s equilibration period was observed prior to analysis.

The rheological measurements were carried using two Fann co-axial cylinder viscometers with different torsion spring constants and a Haake co-axial cylinder viscometer with two different bobs (inner cylinders). A broad range of viscosities could be covered using these devices. The relevant dimensions of the viscometers are given in Table 1. Note that in the Fann viscometer, the outer cylinder is rotated while the inner cylinder is kept stationary, whereas in the Haake viscometer, it is the inner cylinder that rotates, and the outer cylinder is stationary. In the Fann viscometer, the rotational speed is varied from 0.9 to 600 rpm, whereas in the Haake viscometer, the rotational speed is varied from 0.01 to 512 rpm. The viscosity standards of known viscosities were used to calibrate the viscometers. All the viscosity measurements were carried out at room temperature (≈23 °C).

The photomicrographs of particles (TG hollow spheres and solospheres S-32) were taken using a Zeiss optical microscope with transmitted light. From the photomicrographs, the size distribution and mean diameter of the particles were determined. A dilute suspension of particles in deionized water was prepared for observation under the microscope. 

The pH measurements were carried out using Fisher Scientific accumet AE150 pH meter. The pH meter was equipped with a pH electrode and temperature probe to measure pH and temperature readings, respectively. The pH was controlled by the addition of drops of 1 molar NaOH solution or concentrated HCl solution (37% A.C.S. Reagent) to the suspension. The NaOH solution was added to increase the pH whereas HCl solution was added to lower the pH. To compensate for dilution of suspension caused by the addition of NaOH or HCl, particles were added to keep the concentration of particles constant.

## 3. Results and Discussion

### 3.1. Rheology of NCC Dispersions

**Figure 2:**
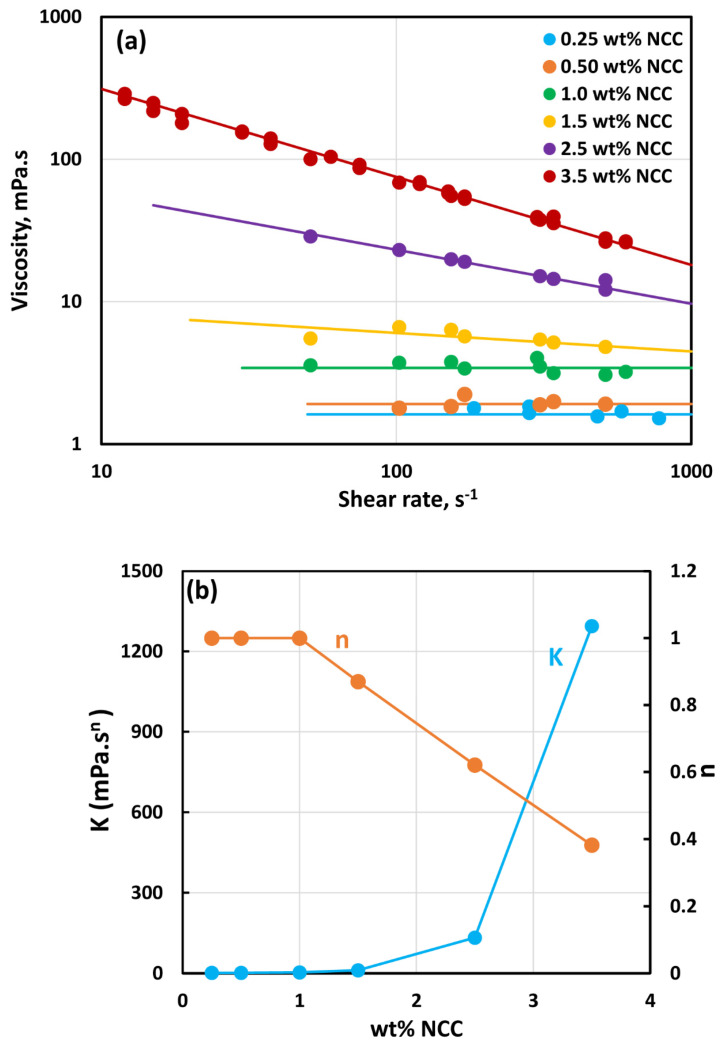
Rheological behavior of NCC dispersions.

Figure 2 shows the rheological data for NCC dispersions. At low concentrations of NCC (≤1.0 wt%), the dispersions are Newtonian with constant viscosity. At higher concentrations, the dispersions are non-Newtonian shear-thinning. The viscosity data follows the power-law model:

(2) $$\tau =K{\dot{\gamma }}^{n}$$

(3) $$\mu =\frac{\tau }{\dot{\gamma }}=K{\dot{\gamma }}^{n-1}$$

where τ is shear stress, $\dot{\gamma }$ is shear rate, K is consistency index, and n is flow behavior index. According to the power-law model (Equation (3)), the viscosity versus shear rate relationship is linear on a log–log plot, as observed in Figure 2a for concentrated NCC dispersions. Figure 2b shows the variations in power-law constants (*K* and *n*) with NCC concentration. The consistency index K rises sharply with the increase in NCC concentration, especially at high concentrations. The flow behavior index n is less than one, indicating shear-thinning behavior. The flow behavior index n decreases with the increase in NCC concentration, indicating an enhancement in shear-thinning with the increase in NCC concentration. These observations are consistent with the literature studies on the rheology of NCC dispersions [27,28].

**Figure 3:**
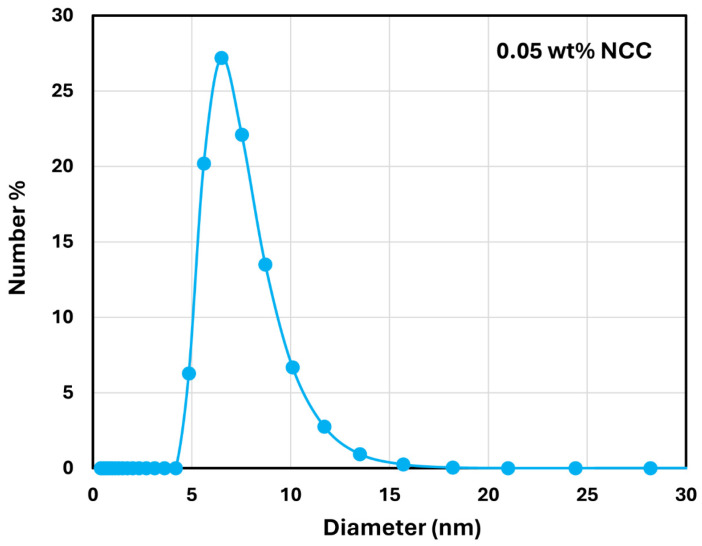
Typical size distribution of NCC.

**Figure 4:**
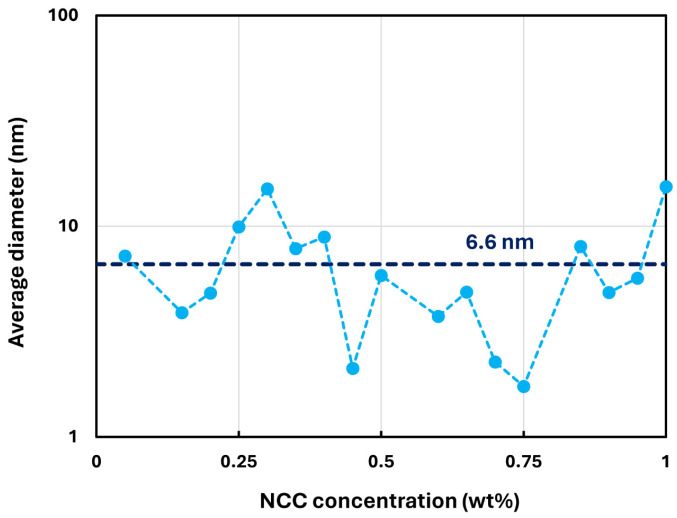
Fluctuation of average hydrodynamic diameter of NCC with concentration.

The shear-thinning behavior in NCC dispersions is due to disaggregation of NCC aggregates and orientation of rod-shaped nanocrystals in the direction of flow [28,29,30,31,32]. The nanocrystals undergo aggregation due to their high surface energy and the presence of van der Waals attractive forces between them. The Brownian motion assists in bringing the nanocrystals into close contact with each other. Figure 3 shows the typical size distribution of nanocrystals as determined by DLS measurement. The number-average hydrodynamic diameter of the NCC fluctuated with the NCC concentration. Figure 4 shows the plot of number average hydrodynamic diameter of NCC as a function of NCC concentration. The overall average hydrodynamic diameter of the nanocrystals is 6.6 nm. 

It should be noted that accurate measurement of the particle size of NCC is difficult due to the following reasons: (a) the particles are rod-shaped (not spherical). The non-spherical shape of particles affects how they scatter light and interact with the imaging technique; (b) NCC tends to aggregate, which can distort the observed size distribution; and (c) NCC consists of a wide range of sizes with varying lengths and widths. 

### 3.2. Rheology of Suspensions of Large Particles (TG Hollow Spheres)

The compositions of suspensions of TG hollow spheres investigated in this study are given in Table 2. The particle concentration ranged from 5 to 50 wt% (6.6 to 57.2 vol%). 

**Figure 5:**
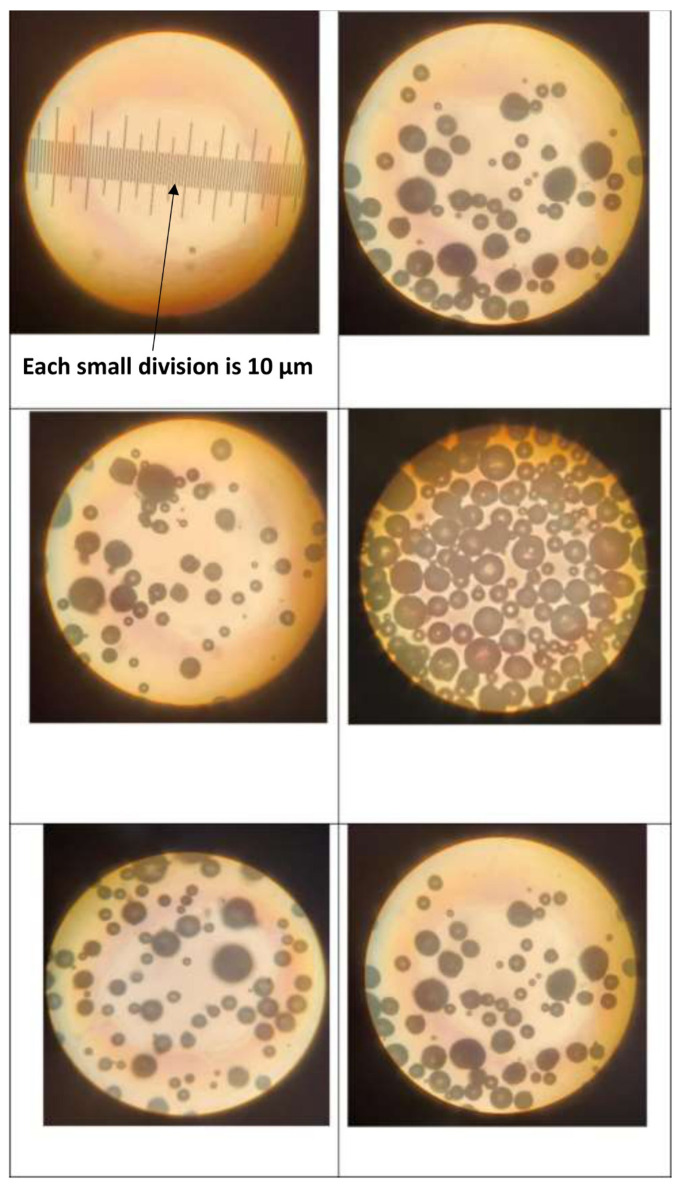
Typical photomicrographs of particles (TG hollow spheres).

**Figure 6:**
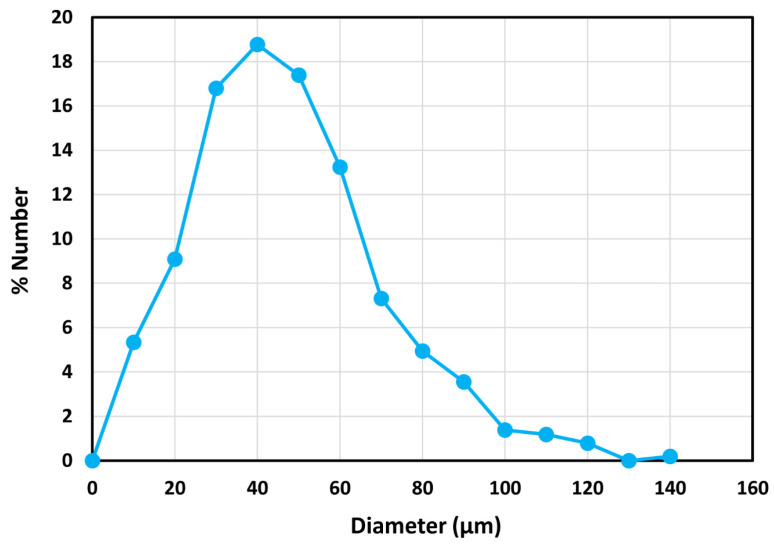
Size distribution of particles (TG hollow spheres).

The typical photomicrographs of particles are shown in Figure 5. The size distribution is shown in Figure 6. Particle size ranged from 10 to 140 μm with a Sauter mean diameter of 69 μm. 

**Figure 7:**
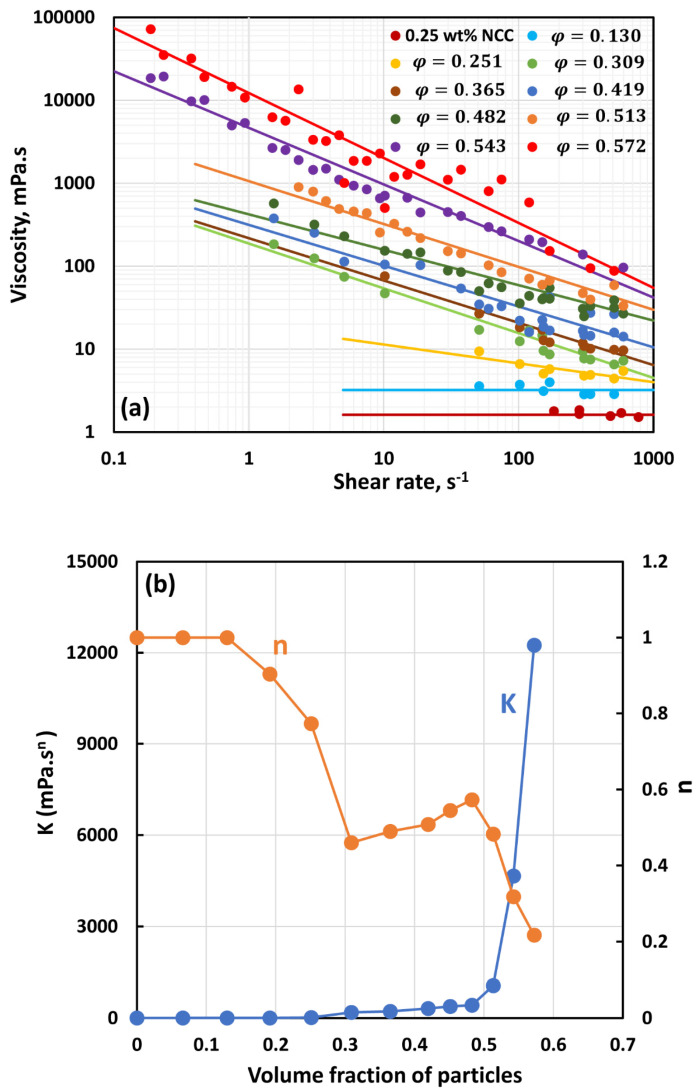
Flow behavior of suspensions at different volume fractions of particles (*φ*) at a fixed NCC concentration of 0.25 wt%.

**Figure 8:**
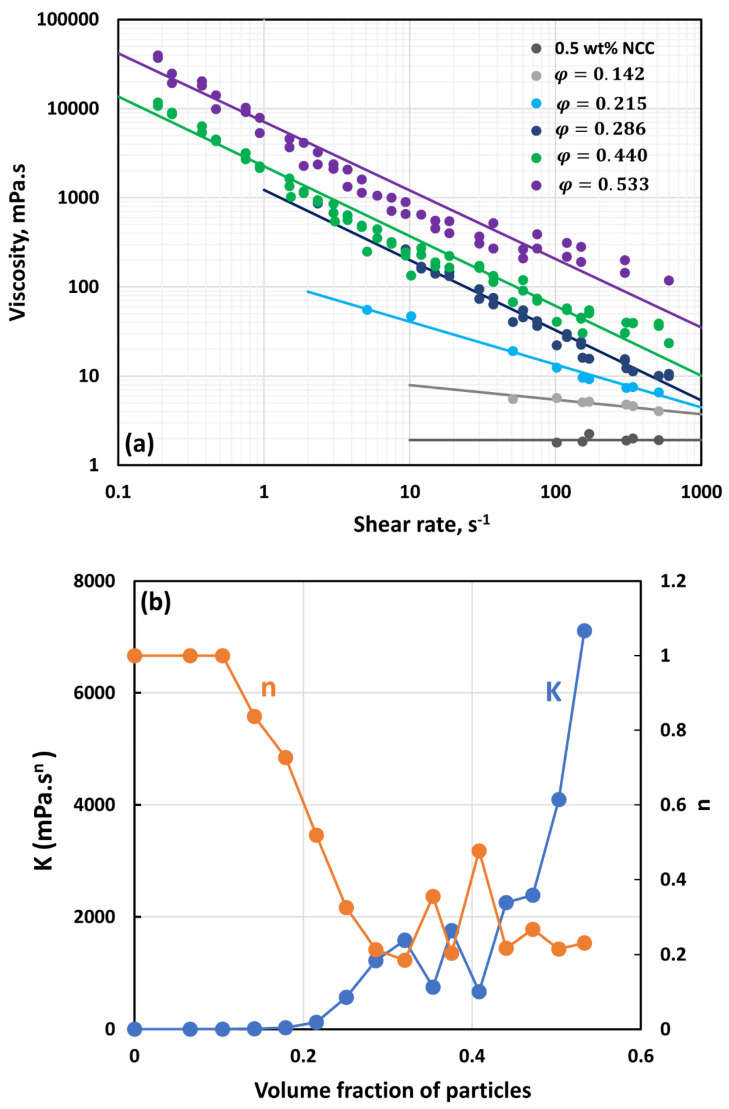
Flow behavior of suspensions at different volume fractions of particles (*φ*) at a fixed NCC concentration of 0.50 wt%.

**Figure 9:**
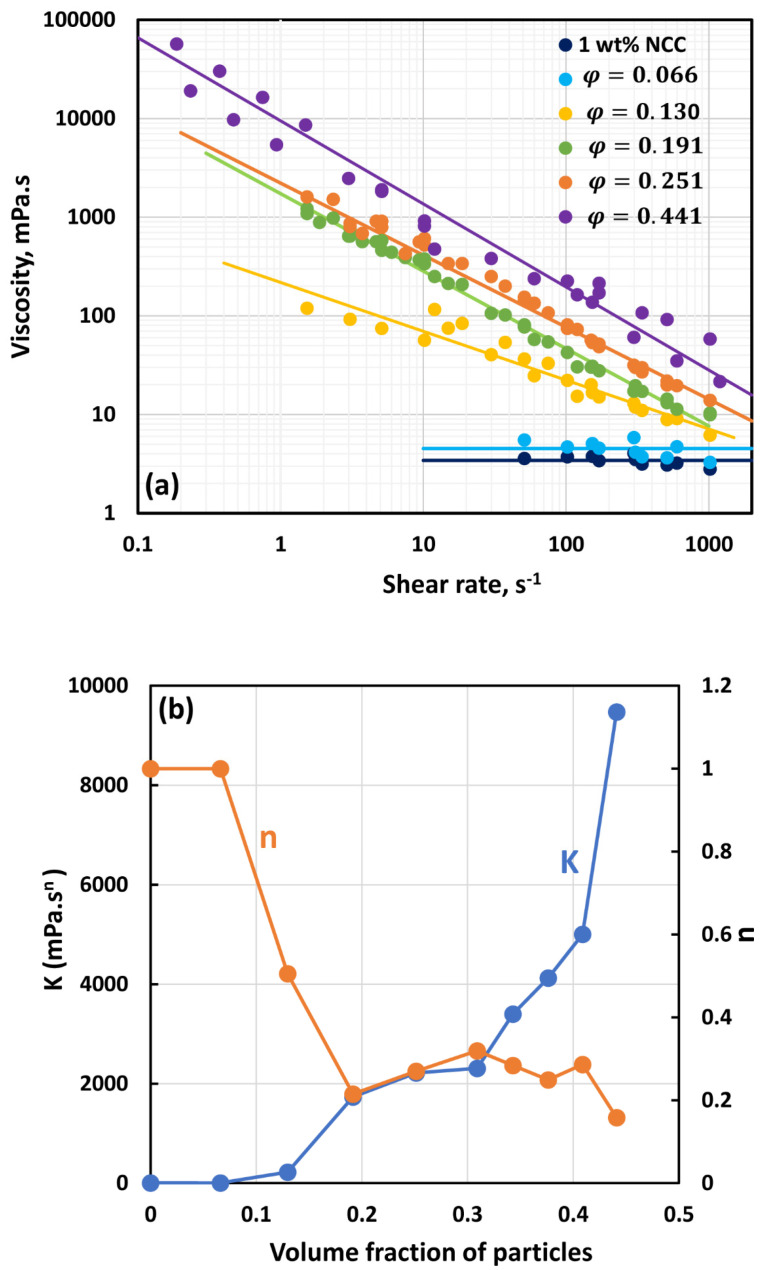
Flow behavior of suspensions at different volume fractions of particles (*φ*) at a fixed NCC concentration of 1.0 wt%.

**Figure 10:**
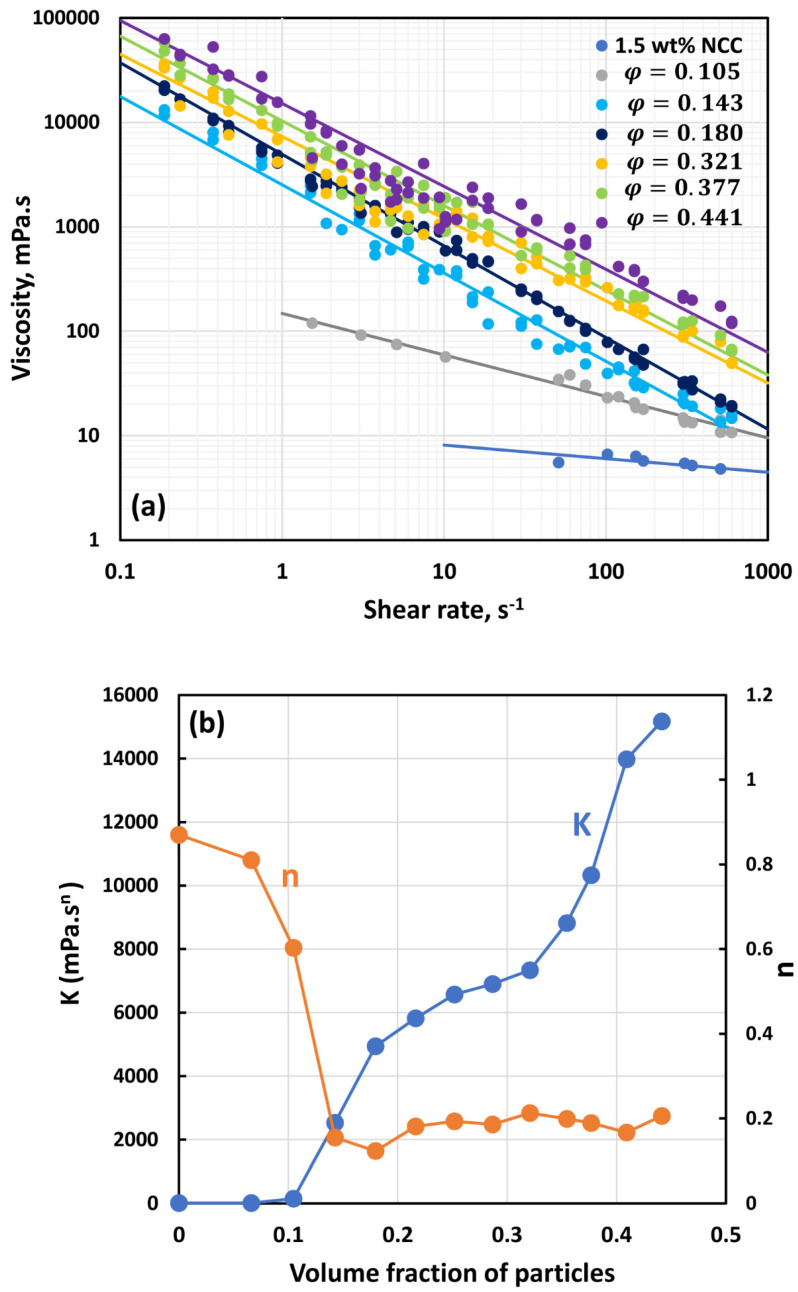
Flow behavior of suspensions at different volume fractions of particles (*φ*) at a fixed NCC concentration of 1.5 wt%.

**Figure 11:**
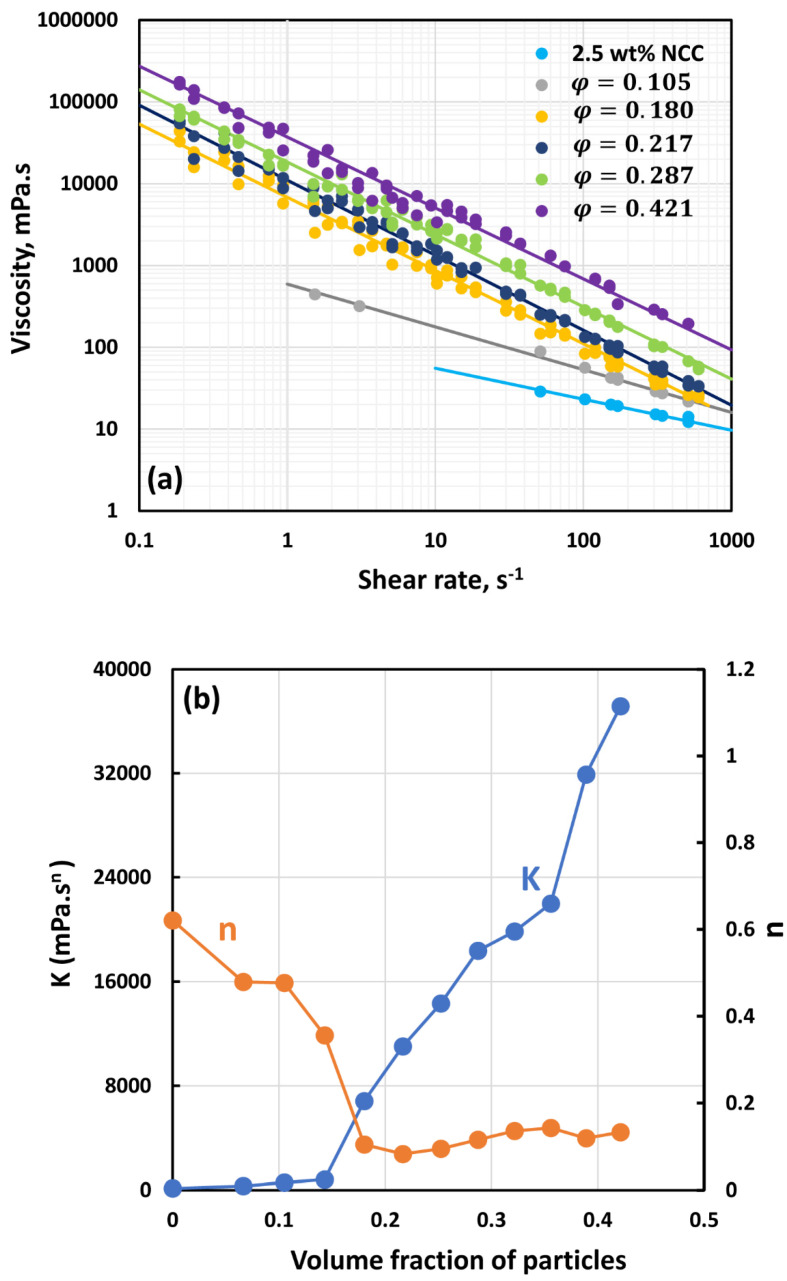
Flow behavior of suspensions at different volume fractions of particles (*φ*) at a fixed NCC concentration of 2.5 wt%.

**Figure 12:**
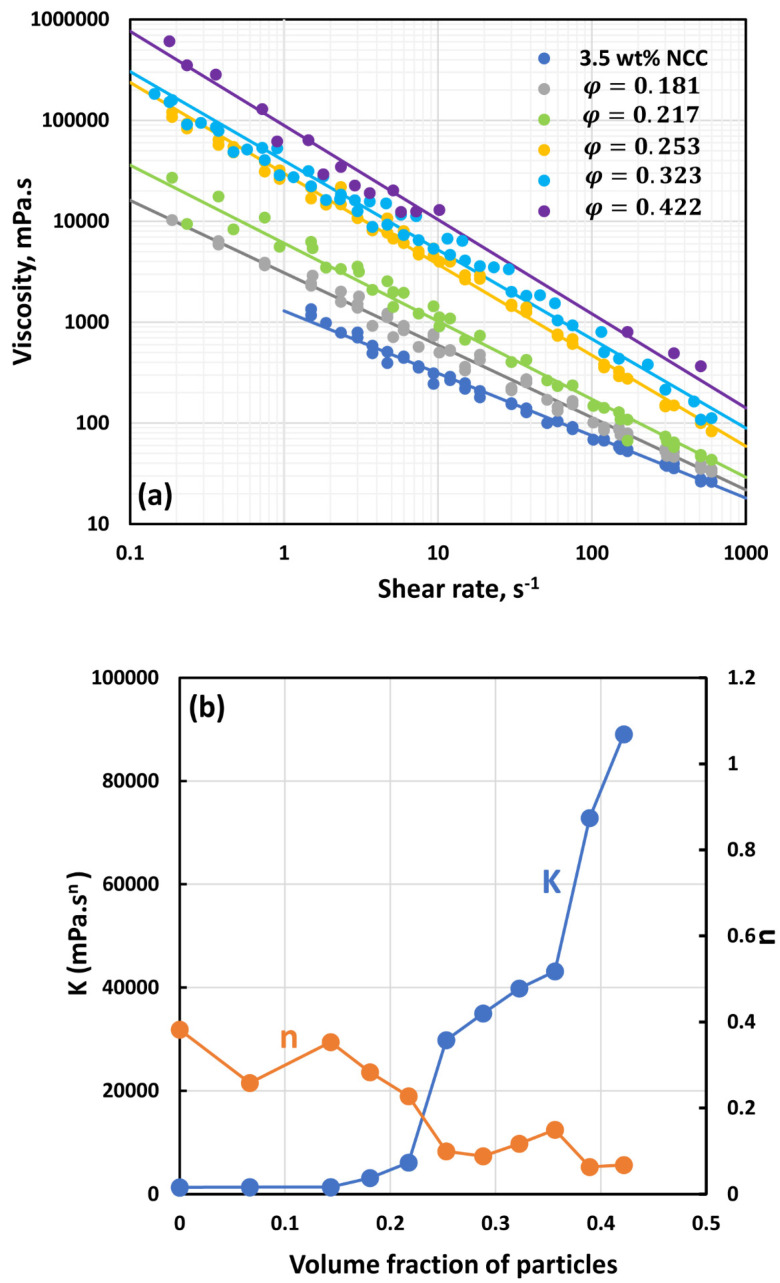
Flow behavior of suspensions at different volume fractions of particles (*φ*) at a fixed NCC concentration of 3.5 wt%.

Figure 7, Figure 8, Figure 9, Figure 10, Figure 11 and Figure 12 show the rheological behavior of suspensions of TG hollow spheres at different volume fractions of particles at a given NCC concentration. The NCC concentration varied from 0.25 to 3.5 wt%. From the figures, the following is clear:

- At low NCC concentrations (≤1 wt%), the suspensions are Newtonian at low volume fractions of particles. At high volume fractions of particles, shear-thinning is observed.
- At high NCC concentrations (≥1.5 wt%), the suspensions are non-Newtonian shear thinning at all volume fractions of particles.
- The degree of shear thinning increases with the increase in particle volume fraction at any given NCC concentration.
- All suspensions at different particle volume fractions and different NCC concentrations follow power-law behavior (see Equations (2) and (3)). The plots of viscosity versus shear rate are linear on a log–log scale.

**Figure 13:**
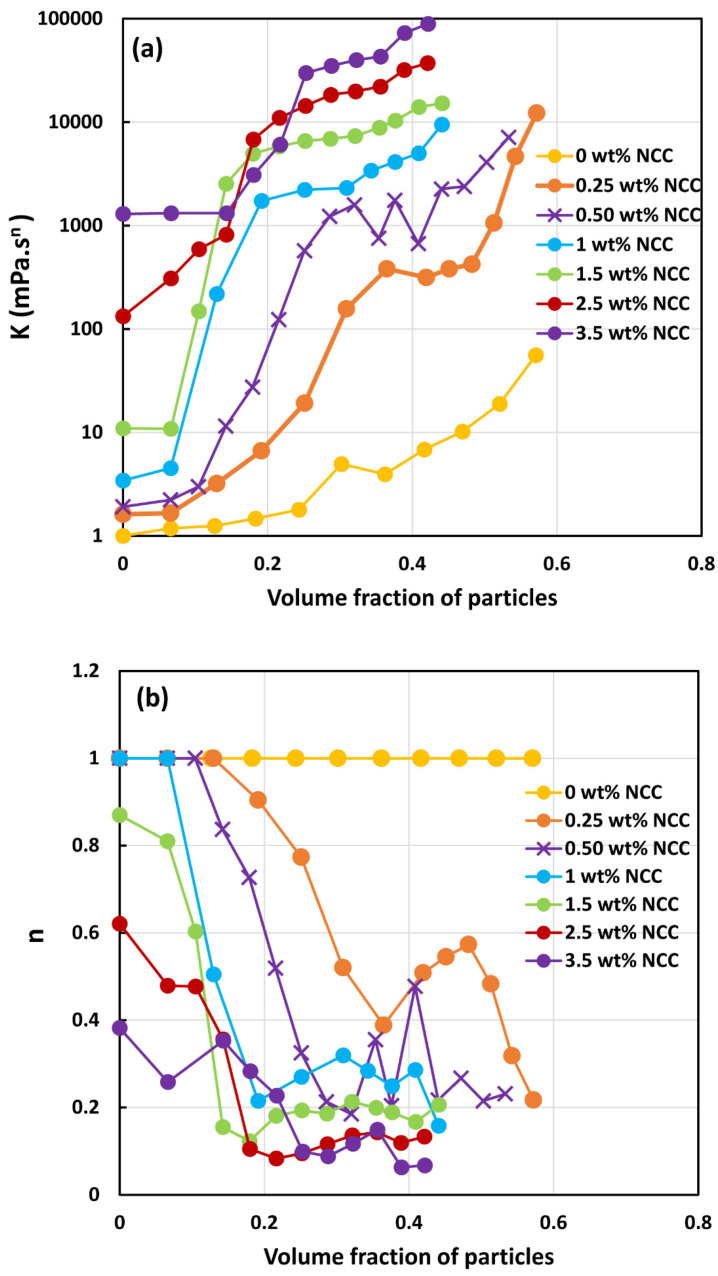
Comparison of power-law constants of suspensions of TG hollow sphere particles at different NCC concentrations: (**a**) consistency index *K*, and (**b**) flow behavior index *n*.

The power-law constants K and n of suspensions were obtained by fitting the power-law model to the viscosity data. Figure 13 compares the power-law constants of suspensions. Figure 13a shows consistency index K as a function of particle volume fraction at different concentrations of NCC, whereas Figure 13b shows flow behavior index n as a function of particle volume fraction at different concentrations of NCC. Figure 13 reveals the following characteristics of the suspensions:

- At a given NCC concentration, the consistency of suspensions as measured by consistency index *K* increases with the increase in particle volume fraction.
- At a fixed particle volume fraction, the consistency of suspension generally increases with the increase in NCC concentration.
- The suspensions containing NCC are shear-thinning (*n* < 1) at a high volume fraction of particles. The flow behavior index *n* depends on both NCC and particle concentrations. It generally decreases with the increase in NCC and particle concentrations.

**Figure 14:**
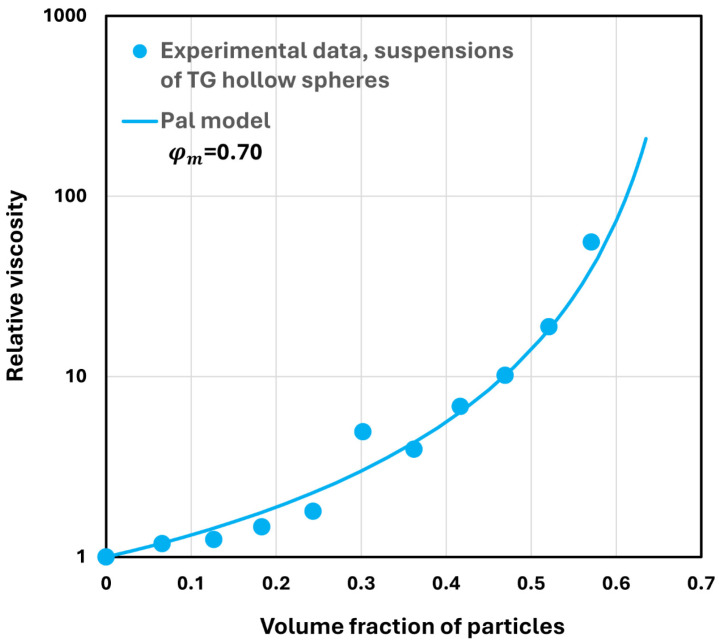
Comparison of experimental viscosity data with model prediction.

As indicated in Figure 13b, the suspensions of TG hollow spheres without NCC are Newtonian (n=1) over the full range of volume fraction of particles investigated. As the viscosity of Newtonian suspensions has been modelled extensively in the available literature on suspension rheology [33], it is worthwhile to compare the experimental data for suspensions for TG hollow spheres with the predictions of the literature models. Pal [33] recently proposed the following viscosity model for suspensions:

(4) $$\eta_{r}={\left[1-\left\{1+\left(\frac{1-\varphi_{m}}{\varphi_{m}^{2}}\right)\varphi \right\}\varphi \right]}^{-2.5}$$

where $\eta_{r}$ is the relative viscosity of suspension, φ is volume fraction of particles, and $\varphi_{m}$ is the maximum packing volume fraction of particles. This model describes the viscosity data accurately for concentrated suspensions of unimodal hard spheres. Based on a large body of experimental data on monodisperse suspensions, $\varphi_{m}$ was found to be 0.632 [33]. Figure 14 shows comparison between the experimental data and model predictions. There is excellent agreement between the model predictions and experimental data. However, the maximum packing volume fraction $\varphi_{m}$ is significantly higher than 0.632. A high $\varphi_{m}$ value of 0.70 indicates that the suspension is polydisperse, as observed experimentally (see Figure 6).

### 3.3. Rheology of Suspensions of Small Particles (Solospheres S-32)

The compositions of suspensions of solospheres S-32 investigated in this study are given in Table 3. The particle concentration ranged from 4.7 to 65 wt% (2.3 to 46.7 vol%). 

**Figure 15:**
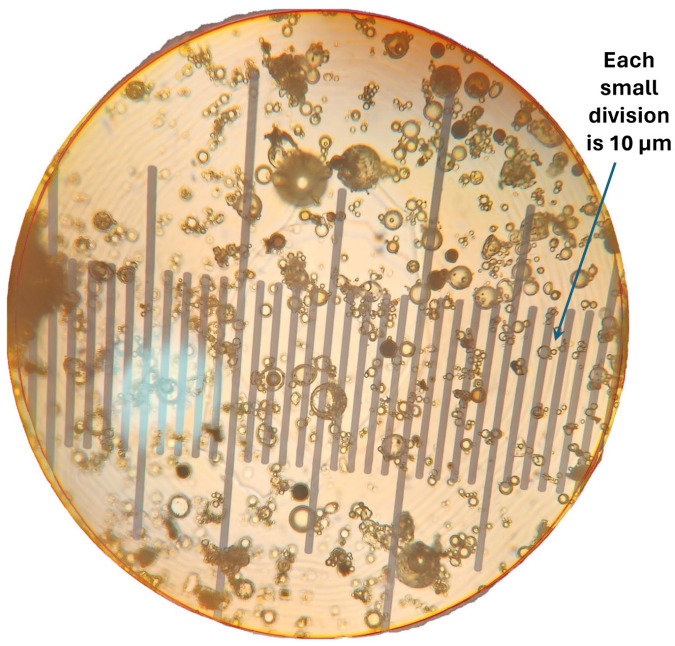
Typical photomicrograph of solospheres S-32 particles.

**Figure 16:**
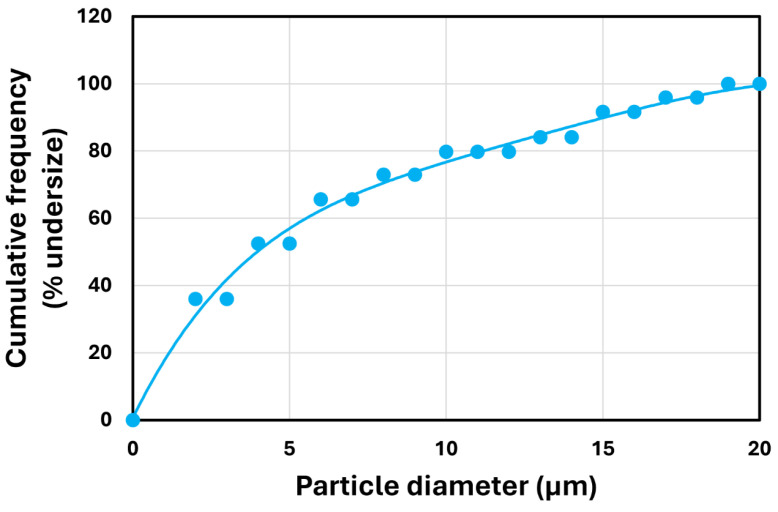
Particle size distribution of solospheres S-32 particles.

The typical photomicrograph of solospheres S-32 particles is shown in Figure 15. The size distribution is shown in Figure 16. The particle diameter ranged from approximately 2 to 20 μm with a Sauter mean diameter of 14 μm. More than 1000 particles were counted to determine the particle size distribution and the mean diameter. Note that the solospheres S-32 particles are much smaller in size as compared with TG hollow sphere particles. The Sauter mean diameter of the TG hollow spheres was five times that of the solospheres S-32. 

**Figure 17:**
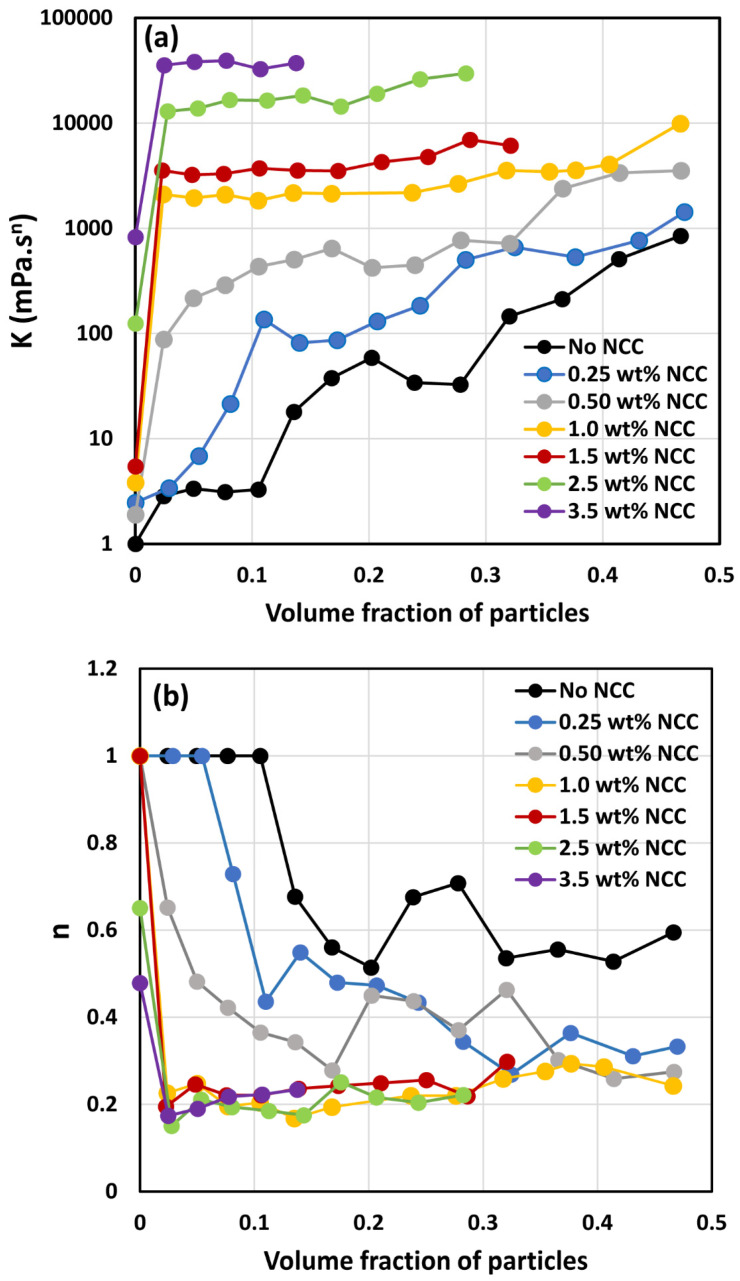
Comparison of power-law constants of suspensions of solospheres S-32 particles at different NCC concentrations: (**a**) consistency index *K*, and (**b**) flow behavior index *n*.

Like suspensions of TG hollow sphere particles, the suspensions of solospheres S-32 particles also followed the power-law behavior; that is, the plots of viscosity versus shear rate were linear on log–log scale and the data could be fitted by the power-law model (see Equations (2) and (3)). Figure 17 compares the power-law parameters (K and n) for suspensions of solospheres containing different concentrations of NCC. The consistency index increases with the increase in particle volume fraction at a given NCC concentration. The consistency index also increases with the increase in NCC concentration at a fixed particle volume fraction. At a high volume fraction of particles, the suspensions are shear-thinning (n<1). The degree of shear-thinning increases with the increases in particle volume fraction and NCC concentration. It should be noted that most of the changes occur initially at low volume fractions of particles and then the power-law parameters tend to level off at high particle volume fractions. 

**Figure 18:**
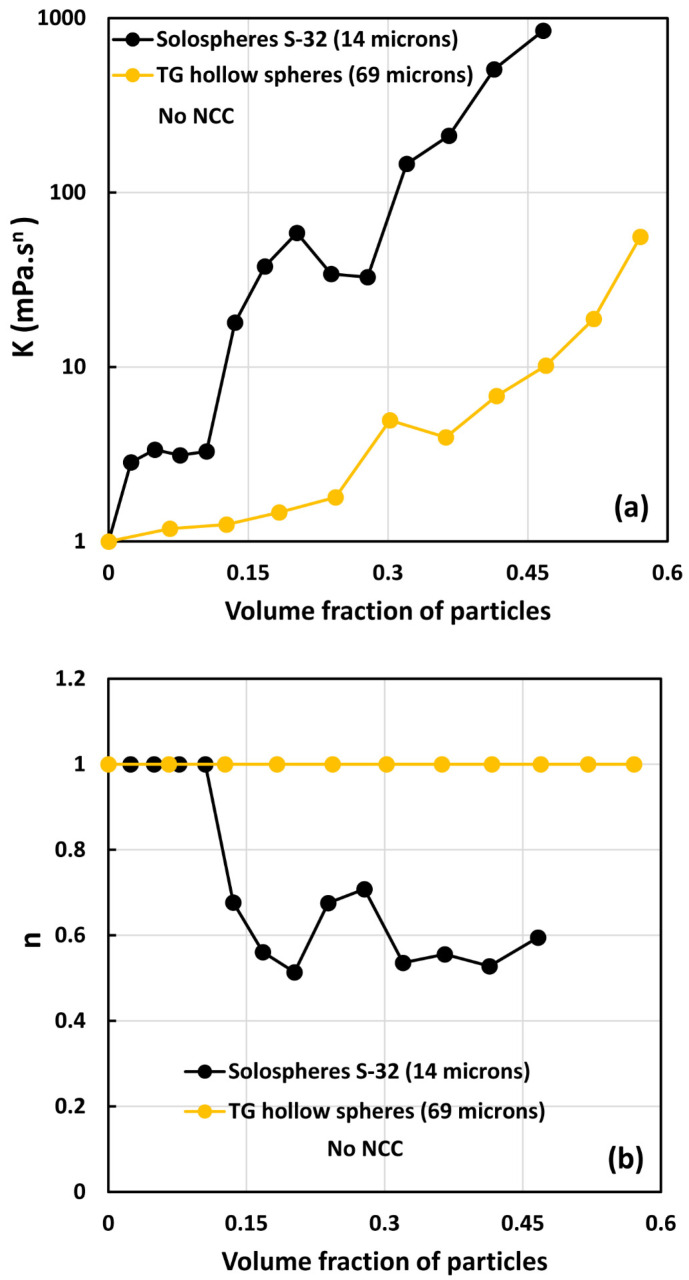
Comparison of power-law constants of suspensions of solospheres S-32 particles with suspensions of TG hollow spheres with no NCC: (**a**) consistency index *K*, and (**b**) flow behavior index *n*.

Figure 18 compares the consistency index K and flow behavior index n of the two types of suspensions investigated, that is, TG hollow sphere suspensions and solosphere S-32 suspensions in the absence of any NCC addition. The suspensions of small particles (solospheres S-32) are much more viscous than the large-sized TG hollow sphere suspensions (see Figure 18). Interestingly, the suspensions of the small-sized particles are also non-Newtonian shear-thinning (n<1) whereas the suspensions of large-sized particles are Newtonian over the full range of particle volume fraction investigated. This clearly indicates the suspensions of small particles form aggregates of particles, especially when the volume fraction of particles is larger than 0.1, whereas the suspensions of large particles are uniformly dispersed with negligible aggregation at all concentrations investigated. Note that according to the available literature on the rheology of suspensions, suspensions of inert non-Brownian spherical particles dispersed in a Newtonian matrix generally behave as Newtonian fluids [6,10,13,34,35,36]. Furthermore, the effect of particle size on suspension rheology is negligible. However, at high particle concentrations close to maximum packing concentration, the particle size distribution has a strong effect on the rheological properties. The rheological properties decrease with the increase in polydispersity of suspensions [34,37]. 

**Figure 19:**
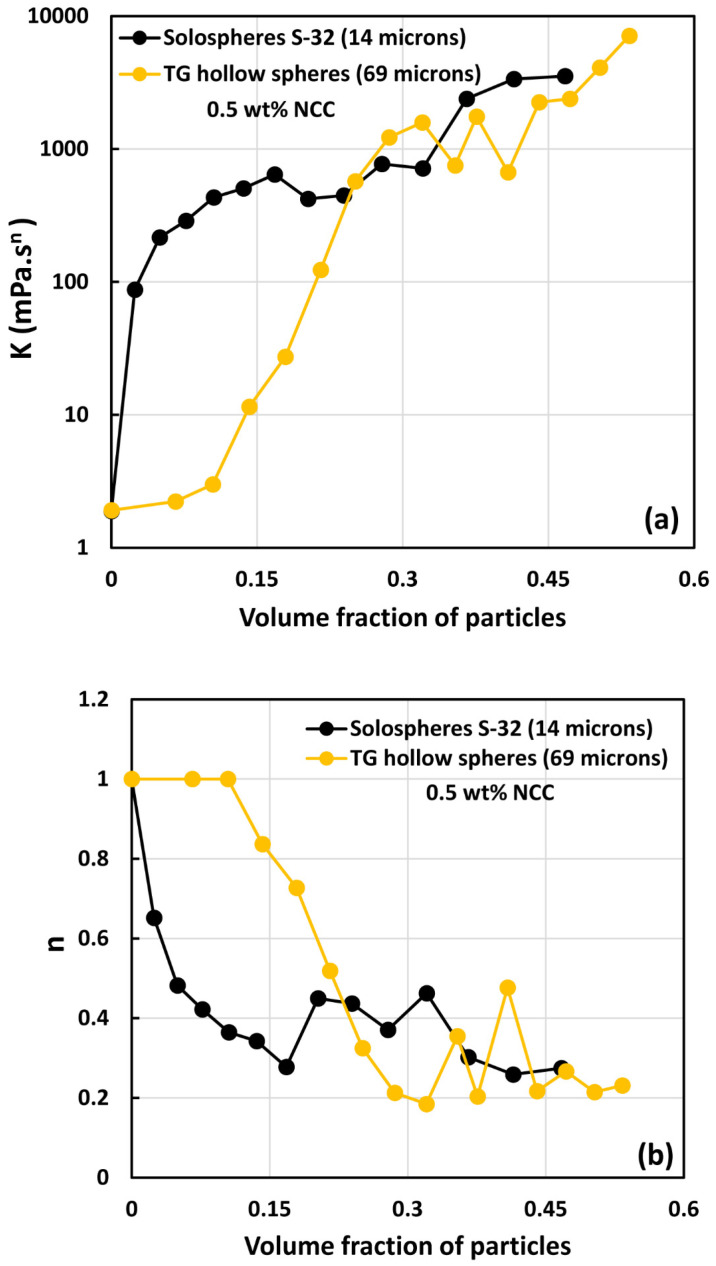
Comparison of power-law constants of suspensions of solospheres S-32 particles with suspensions of TG hollow spheres at NCC concentration of 0.5 wt%: (**a**) consistency index *K*, and (**b**) flow behavior index *n*.

**Figure 20:**
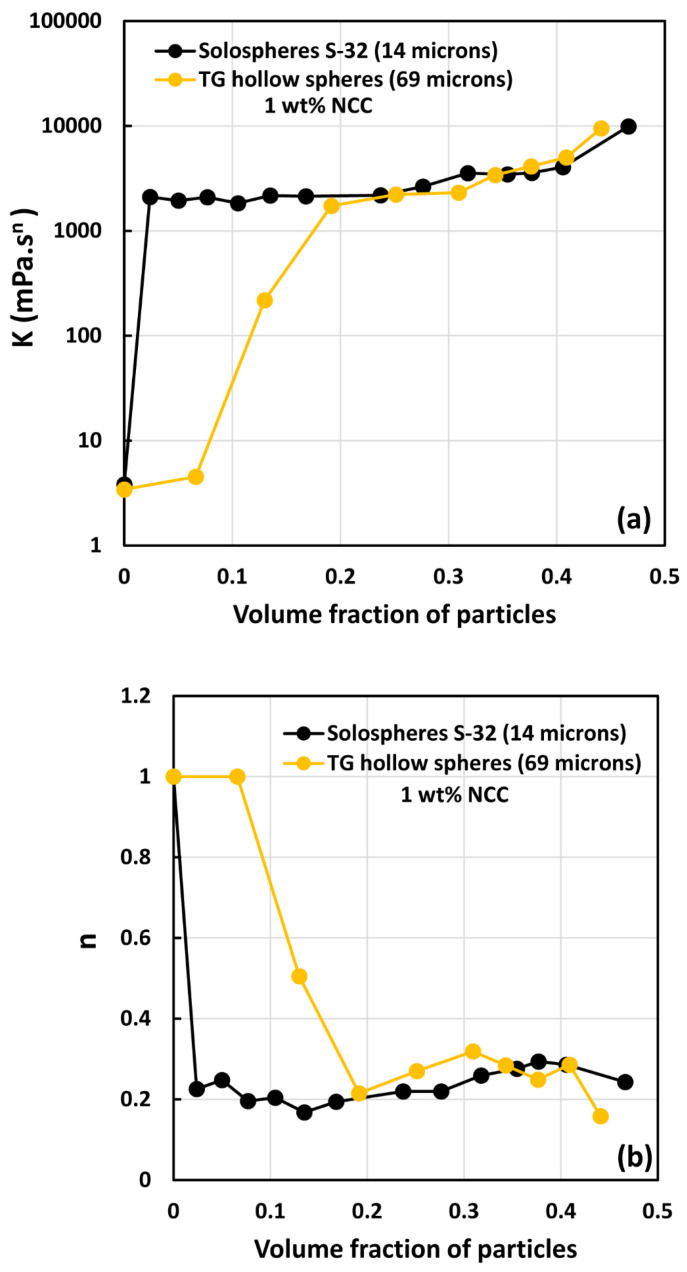
Comparison of power-law constants of suspensions of solospheres S-32 particles with suspensions of TG hollow spheres at NCC concentration of 1 wt%: (**a**) consistency index *K*, and (**b**) flow behavior index *n*.

**Figure 21:**
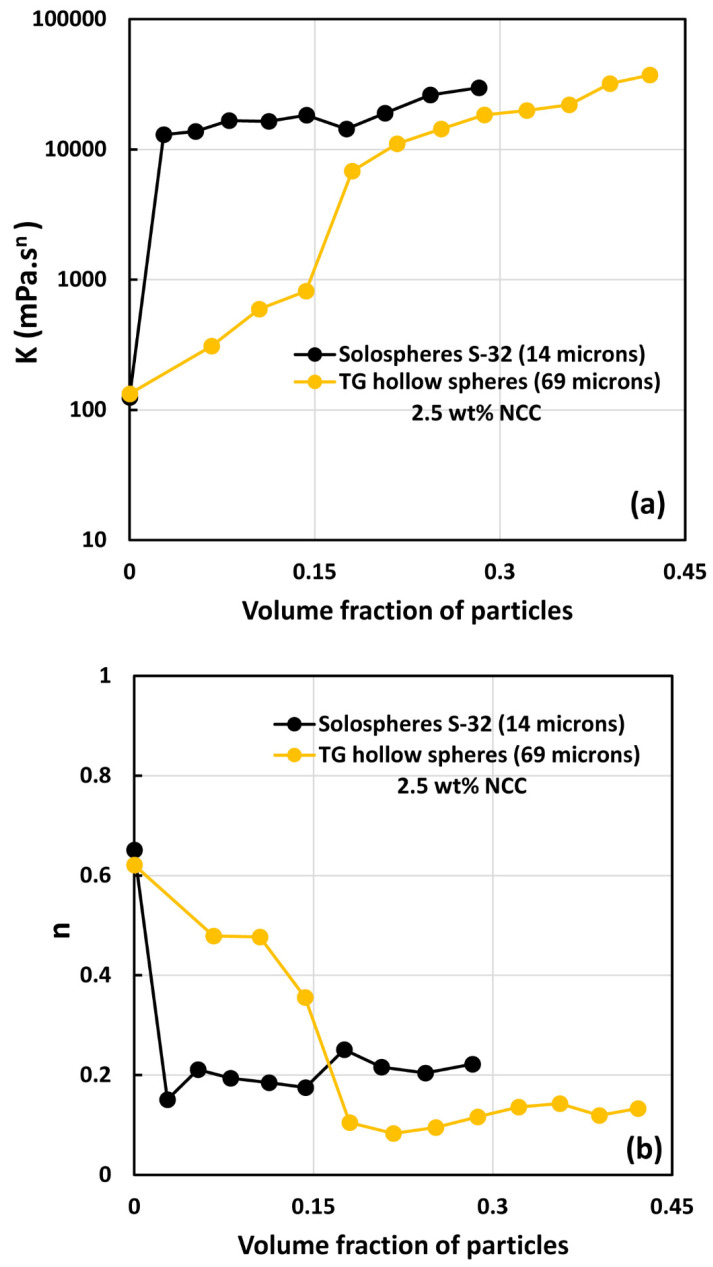
Comparison of power-law constants of suspensions of solospheres S-32 particles with suspensions of TG hollow spheres at NCC concentration of 2.5 wt%: (**a**) consistency index *K*, and (**b**) flow behavior index *n*.

Figure 19, Figure 20 and Figure 21 compare the consistency index K and flow behavior index n of suspensions of TG hollow spheres and solospheres S-32 in the presence of NCC at different NCC concentrations. Interestingly, at low volume fractions of particles, the suspensions of small particles (solospheres S-32) are still more viscous and shear-thinning than the suspensions of large particles (TG hollow spheres). However, at high volume fraction of particles, both the consistency index K and flow behavior index n of the two types of suspensions (small and large particles) overlap. This clearly indicates that NCC is playing a role in causing aggregation of particles. At high volume fraction of particles, both small sized and large sized suspensions are aggregated due to the presence of NCC and hence the rheological properties of the two suspension systems overlap.

### 3.4. Effect of Salt on the Rheology of Suspensions

**Figure 22:**
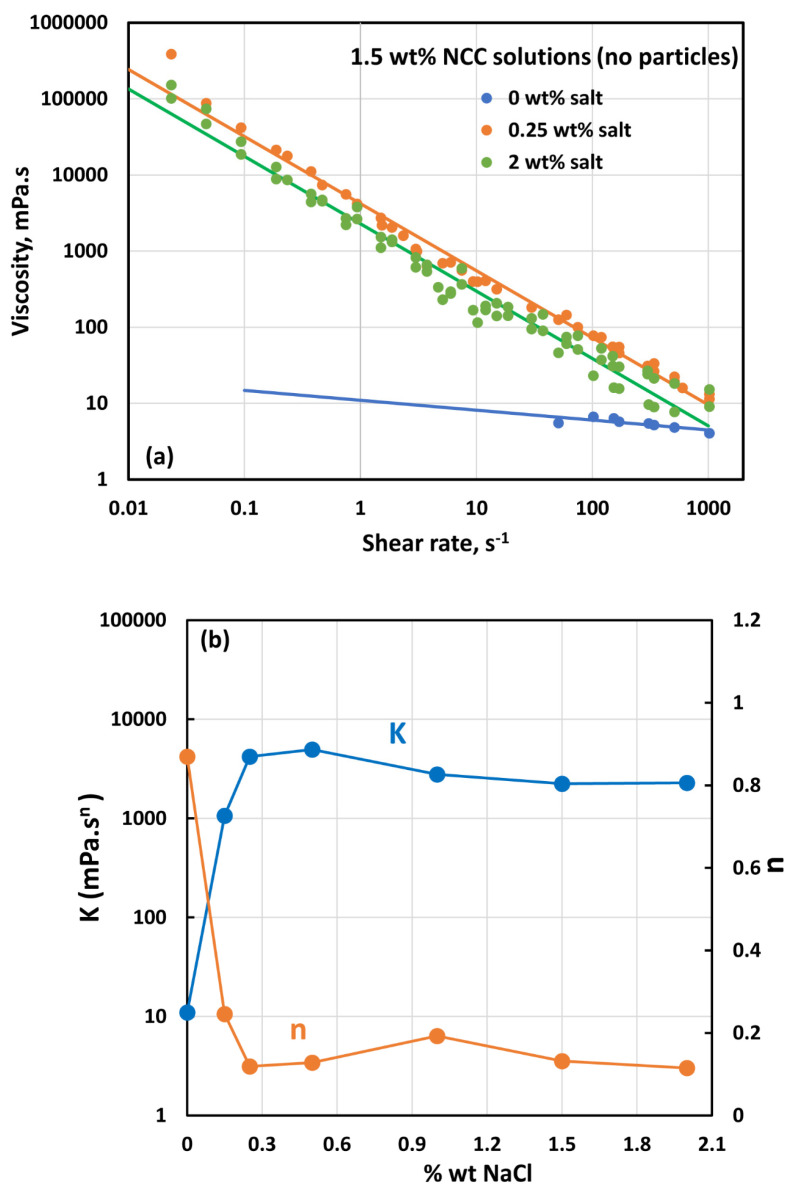
Effect of salt (NaCl) on the rheology of 1.5 wt% NCC dispersion without any particles: (**a**) viscosity versus shear rate behavior, and (**b**) variation in consistency index *K* and flow behavior index *n* with salt concentration.

The effect of salt (NaCl) addition on the rheology of NCC solutions (1.5 wt% NCC, without particles) is shown in Figure 22. The NCC solutions become very viscous and highly shear-thinning upon the addition of salt. Note that a sharp jump in the viscosity occurs upon addition of 0.25 wt% salt. With further increase in salt concentration, the rheological properties tend to level off. The influence of salt addition on the power-law parameters is shown in Figure 22b. After an initial jump in K and a sharp fall in n with the addition of salt, the power-law parameters level off with further increase in salt concentration. 

**Figure 23:**
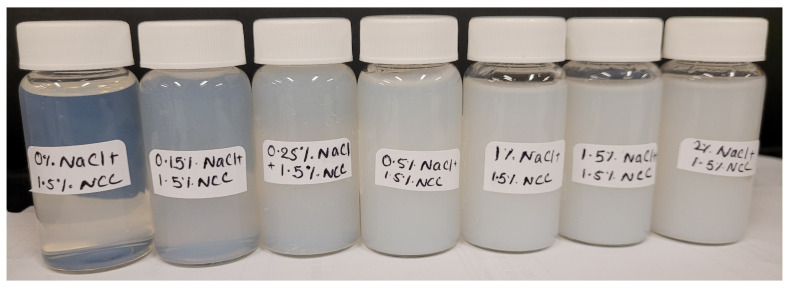
Appearance of 1.5 wt% NCC dispersion samples with the addition of salt (NaCl).

Upon the addition of salt to NCC dispersion, the NCC dispersion tends to gel. The photographs of samples of 1.5 wt% NCC dispersion at different salt concentrations are shown in Figure 23. The solution is very clear at 0% salt but becomes milky with the increase in salt concentration due to the aggregation of the nanocrystals.

**Figure 24:**
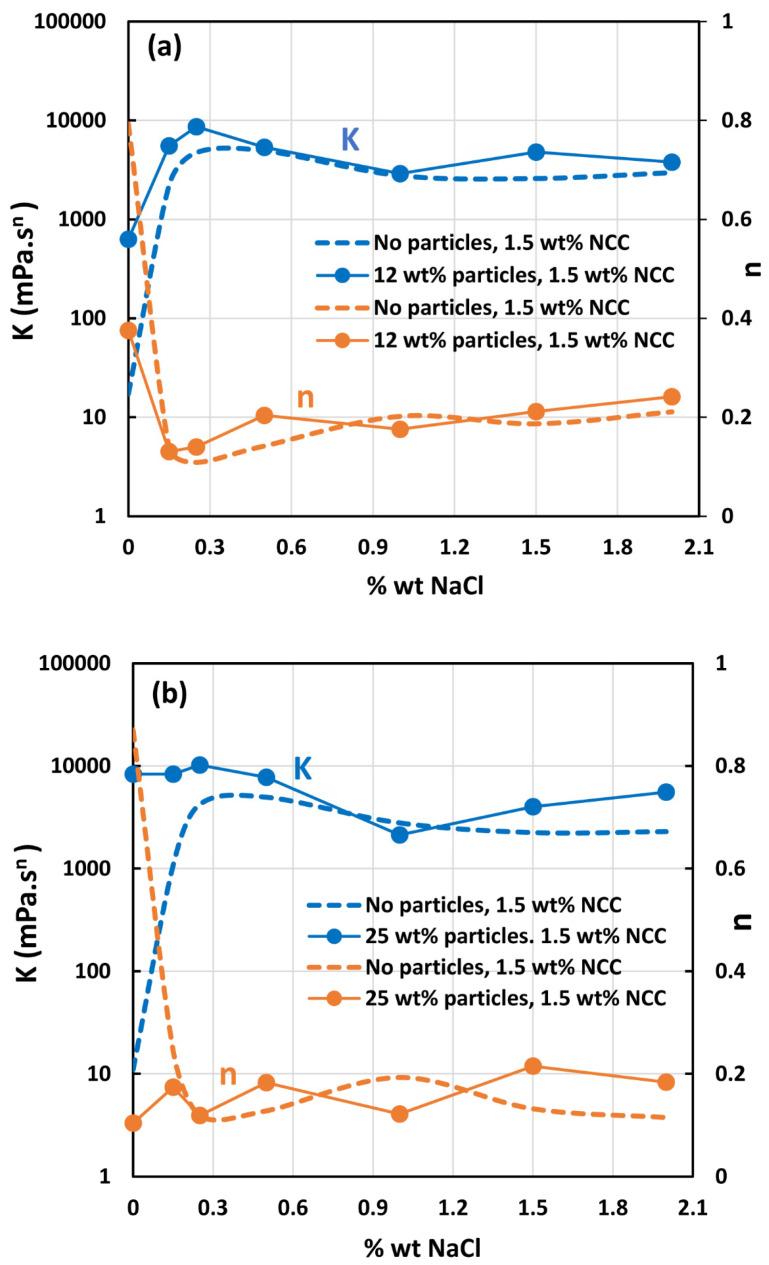
Effect of salt on the power-law constants of suspensions of TG hollow spheres.

**Figure 25:**
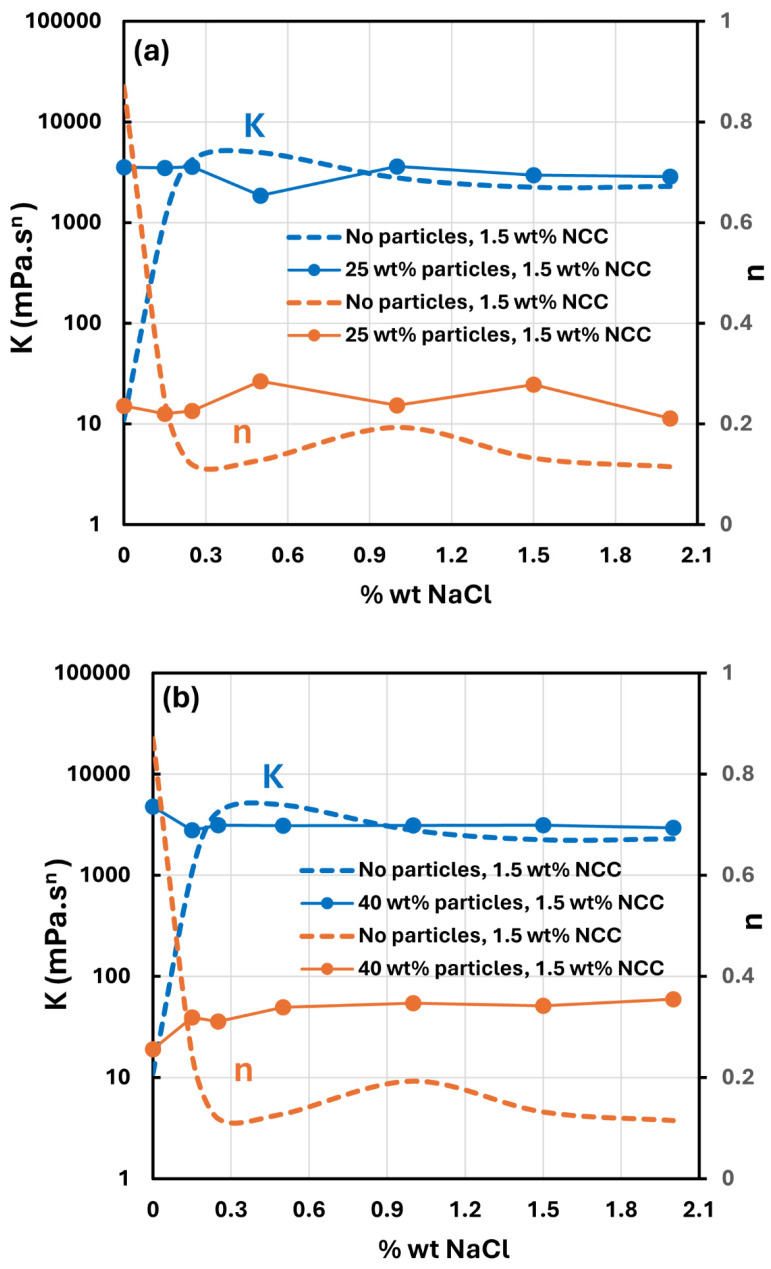
Effect of salt on the power-law parameters of suspensions of solospheres S-32.

Figure 24 shows the effect of salt addition on the rheology of suspensions of TG hollow spheres at two particle concentrations (12 and 25 wt%). The power law parameters consistency index K and flow behavior index n are plotted as a function of salt concentration. For comparison purposes, the power law parameters for NCC dispersion (1.5 wt% NCC) without any particles are also plotted. Interestingly the addition of salt has little effect on the rheology of suspensions thickened by NCC. Furthermore, the power law parameters of suspensions are almost the same as that of the NCC dispersion without any particles at salt concentrations higher than 0.3 wt%. A similar behavior is exhibited by suspensions of solospheres S-32 as shown in Figure 25.

### 3.5. Effect of pH on the Rheology of Suspensions

**Figure 26:**
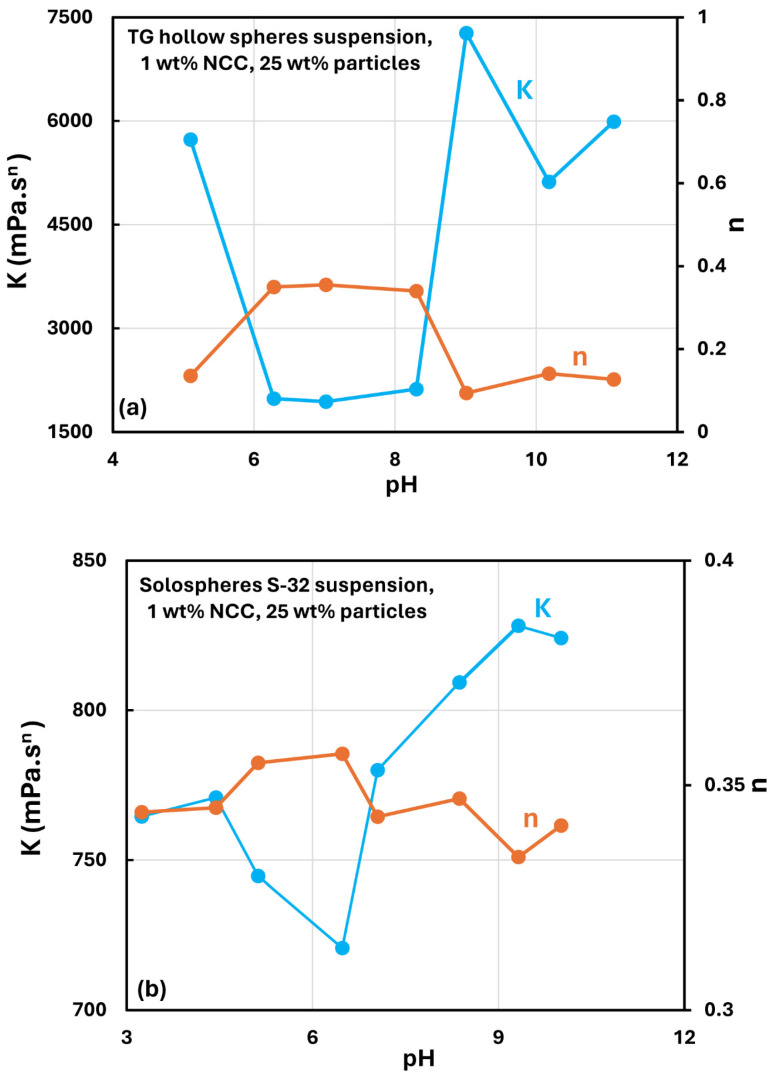
Effect of pH on the power-law parameters of suspensions: (**a**) suspensions of TG hollow spheres, and (**b**) suspensions of solospheres S-32.

Figure 26 shows the effect of pH on the rheological behavior of suspensions. Both types of suspensions (TG hollow spheres and solospheres S-32) show a minimum in the consistency index K under neutral conditions (pH ≅ 7). The consistency of the suspension increases under acidic or alkaline conditions. This observation is consistent with the work of Qi et al. [32], who investigated NCC dispersions without any solid particles. They found that the nanocrystals of NCC undergo severe aggregation under acidic or alkaline conditions, causing a large increase in the viscosity of NCC dispersions. 

### 3.6. Reliability of Rheological Measurements

**Figure 27:**
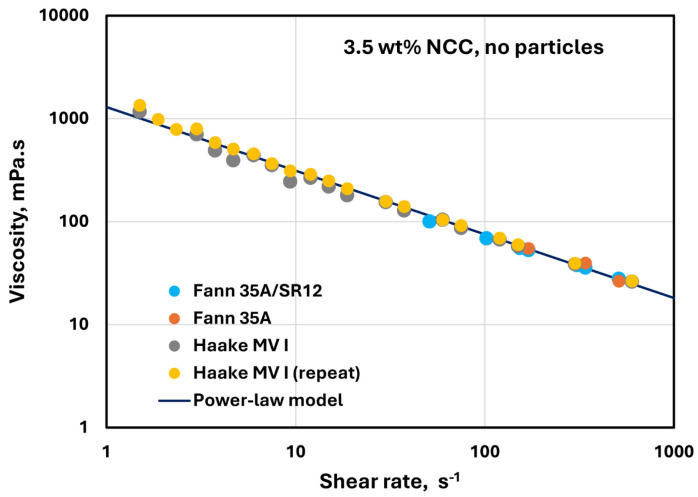
Viscosity data for 3.5 wt% NCC dispersion (no particles) obtained from different viscometers with different gap widths.

The rheological data reported in this work were measured carefully. The data were reproducible and consistent using different viscometers, as described in the Materials and Methods Section (see Section 2.4). Furthermore, there were no wall or slip effects observed in the measurements. For example, Figure 27 shows the data for 3.5 wt% NCC dispersion without any solid particles. The experimental data obtained from the two Fann viscometers and Haake viscometer overlap. Note that the gap width in the Fann and Haake viscometers are different. Furthermore, the repeat test using the Haake viscometer reproduces the data, and all the data could be described by a power-law model accurately. 

**Figure 28:**
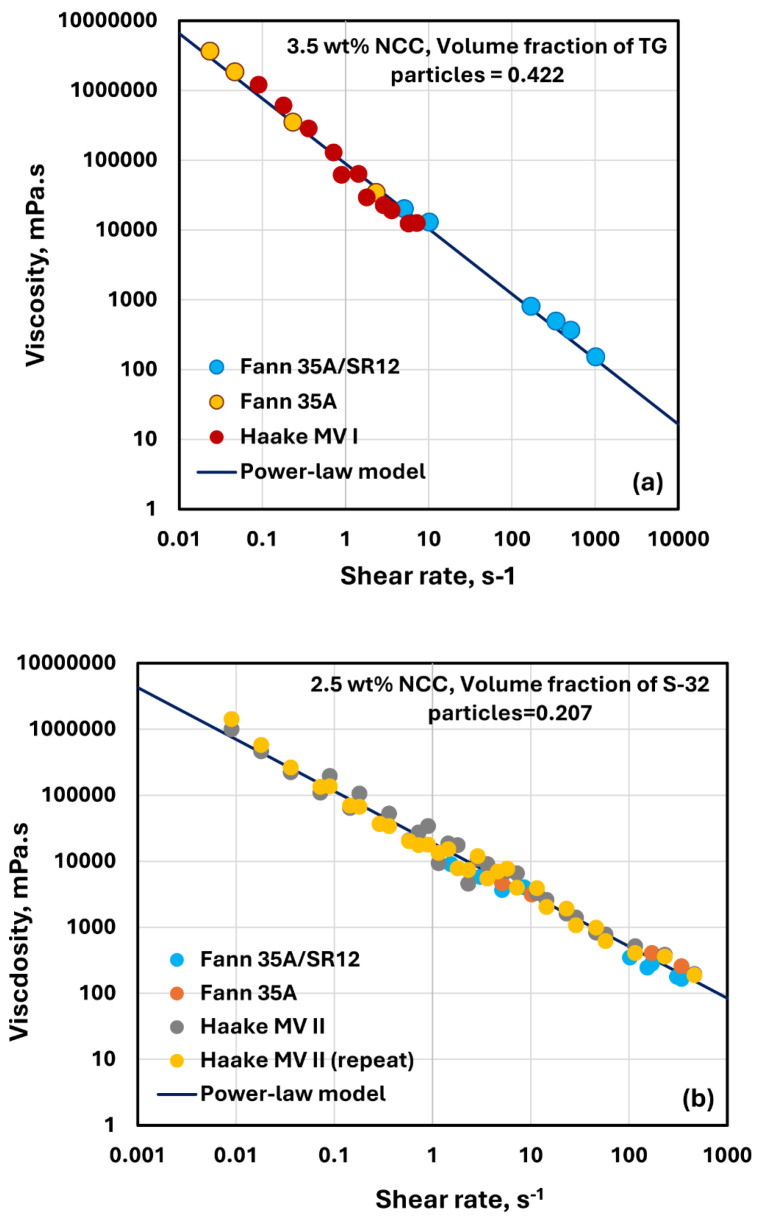
Viscosity versus shear rate data for suspensions obtained from different devices: (**a**) suspension of TG hollow spheres at 3.5 wt% NCC and volume fraction of particles of 0.422; (**b**) suspension of solospheres S-32 at 2.5 wt% NCC and volume fraction of particles of 0.207.

Figure 28 shows sample experimental data for TG hollow sphere and solosphere S-32 suspensions obtained from two Fann viscometers and a Haake viscometer. Figure 28a shows the data for TG hollow sphere suspension with 3.5 wt% NCC and volume fraction of particles of 0.422. Clearly, the data obtained from different devices overlap, indicating the absence of any wall or slip effects. Figure 28b shows the data for solospheres S-32 suspension with 2.5 wt% NCC and volume fraction of particles of 0.207. Once again, the data are reproducible and different devices give consistent values. The data can be described accurately by a power-law model. 

**Figure 29:**
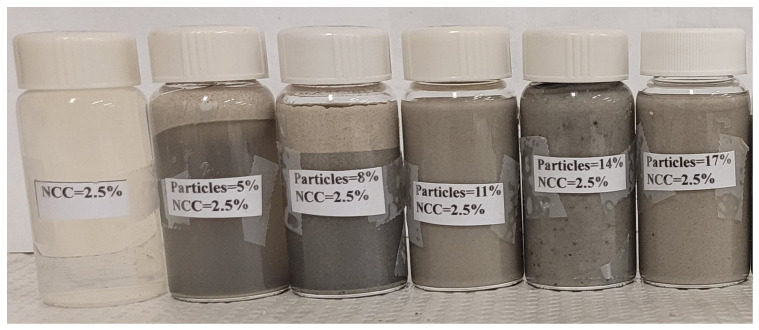
Creaming in suspensions of light hollow spheres at different particle concentrations (wt%) at a fixed NCC concentration of 2.5 wt%. The suspensions were left on the shelf for several months.

The suspensions were homogeneous during the measurements. No creaming (upward rise of light TG hollow particles) or sedimentation (downward settling of heavy solospheres S-32) was detected during the measurements. The addition of NCC to the matrix phase of the suspensions suppressed the creaming and sedimentation of solid particles. Although no systematic studies on the creaming and sedimentation of particles were carried out, the suspension samples exhibited little creaming/sedimentation when left on the shelf for several months, especially at non-dilute concentrations of particles. For example, Figure 29 shows the pictures of suspensions of TG hollow particles when left on the shelf for several months. The NCC concentration of the suspensions is 2.5 wt% based on the matrix phase. Creaming of light particles (TG hollow spheres) is observed only at low particle concentrations of 5 and 8 wt%. At higher particle concentrations, no creaming occurs even after several months. 

## 4. Conclusions

The viscous behavior of suspensions thickened by cellulose nanocrystals (referred to as NCC) was investigated experimentally. The effects of cellulose nanocrystal concentration, particle concentration, particle size, salt concentration, and pH on the viscous behavior of suspensions were determined. Based on the experimental work, the following conclusions can be made:

- The dispersions of cellulose nanocrystals at NCC ≥ 1 wt% are shear-thinning due to disaggregation and orientation of nanocrystals with shear.
- The suspensions of large-size particles (TG hollow spheres, Sauter mean diameter 69 µm) are Newtonian in the absence of cellulose nanocrystals. However, the addition of nanocrystals makes them shear-thinning and more viscous. The degree of shear-thinning increases with the increases in NCC and particle concentrations.
- The suspensions of small size particles (solospheres S-32, Sauter mean diameter 14 µm) are shear-thinning at particle volume fractions > 0.1 even in the absence of any nanocrystals. The addition of nanocrystals makes them more shear-thinning and viscous.
- The addition of salt has a strong influence on the rheology of nanocrystal dispersions. A sharp rise in the consistency index and a large drop in the flow behavior index are observed with the addition of salt. However, the addition of salt has little effect on the rheology of suspensions thickened by NCC.
- The nanocrystal-thickened suspensions show a minimum in consistency index under neutral condition (pH ≅ 7). The consistency rises substantially with a decrease in pH below 7 and with an increase in pH above 7.

## Figures and Tables

**Table 1:** Relevant dimensions of viscometers used in this study.

Viscometer	Inner Cylinder Radius, *R_i_*	Outer Cylinder Radius, *R_o_*	Length of Inner Cylinder	Gap Width
Fann 35A/SR-12 (low torsion spring constant)	1.72 cm	1.84 cm	3.8 cm	0.12 cm
Fann 35A (high torsion spring constant)	1.72 cm	1.84 cm	3.8 cm	0.12 cm
Haake Rotovisco RV 12 with MV I	2.00 cm	2.1 cm	6.0 cm	0.10 cm
Haake Rotovisco RV 12 with MV II	1.84 cm	2.1 cm	6.0 cm	0.26 cm

**Table 2:** Compositions of suspensions of TG hollow spheres investigated in this study.

NCC Concentration of Matrix Phase (wt%)	Solids Concentration of Suspension (wt%)	Solids Concentration of Suspension (vol%)
0	Ten concentrations: 5, 9.8, 14.4, 19.4, 24.4, 29.8, 34.8, 39.8, 44.8, 49.8	Ten concentrations: 6.6, 12.7, 18.3, 24.4, 30.2, 36.2, 41.7, 47.0, 52.1, 57.1
0.25	Twelve concentrations: 5, 10, 15, 20, 25, 30, 35, 38, 41, 44, 47, 50	Twelve concentrations: 6.6, 13.0, 19.1, 25.1, 30.9, 36.5, 41.9, 45.1, 48.2, 51.3, 54.3, 57.2
0.50	Fifteen concentrations: 5, 8, 11, 14, 17, 20, 23, 26, 29, 31, 34, 37, 40, 43, 46	Fifteen concentrations: 6.6, 10.4, 14.2, 17.9, 21.5, 25.1, 28.6, 32, 35.4, 37.6, 40.8, 44, 47.2, 50.3, 53.3
1.0	Nine concentrations: 5, 10, 15, 20, 25, 28, 31, 34, 37	Nine concentrations: 6.6, 13.0, 19.1, 25.1, 30.9, 34.3, 37.6, 40.9, 44.1
1.5	Twelve concentrations: 5, 8, 11, 14, 17, 20, 23, 26, 29, 31, 34, 37	Twelve concentrations: 6.6, 10.5, 14.3, 18.0, 21.6, 25.2, 28.7, 32.1, 35.5, 37.7, 40.9, 44.1
2.5	Eleven concentrations: 5, 8, 11, 14, 17, 20, 23, 26, 29.1, 32.1, 35	Eleven concentrations: 6.6, 10.5, 14.3, 18.0, 21.7, 25.2, 28.7, 32.2, 35.6, 38.9, 42.1
3.5	Ten concentrations: 5, 11, 14, 17, 20, 23, 26, 29, 32, 35	Ten concentrations: 6.7, 14.3, 18.1, 21.7, 25.3, 28.8, 32.3, 35.6, 38.9, 42.2

**Table 3:** Compositions of suspensions of solospheres S-32 investigated in this study.

NCC Concentration of Matrix Phase (wt%)	Solids Concentration of Suspension (wt%)	Solids Concentration of Suspension (vol%)
0	Thirteen concentrations: 5, 10, 15, 20, 25, 30, 35, 40, 45, 50, 55, 60, 65	Thirteen concentrations: 2.4, 5.0, 7.7, 10.5, 13.6, 16.8, 20.2, 23.9, 27.8, 32, 36.5, 41.4, 46.6
0.25	Thirteen concentrations: 5.9, 10.9, 15.8, 20.8, 25.7, 30.7, 35.6, 40.6, 45.5, 50.5, 56.2, 61.6, 65.3	Thirteen concentrations: 2.9, 5.4, 8.1, 11.0, 14.0, 17.3, 20.7, 24.4, 28.3, 32.5, 37.7, 43.1, 47
0.50	Thirteen concentrations: 5, 10, 15, 20, 25, 30, 35, 40, 45, 50, 55, 60, 65	Thirteen concentrations: 2.4, 5.0, 7.7, 10.5, 13.6, 16.8, 20.3, 23.9, 27.8, 32.0, 36.6, 41.4, 46.7
1.0	Thirteen concentrations: 4.9, 10, 15, 19.9, 24.8, 29.9, 39.7, 44.7, 49.6, 53.7, 56.1, 59.1, 64.9	Thirteen concentrations: 2.4, 5, 7.7, 10.5, 13.5, 16.8, 23.7, 27.6, 31.8, 35.4, 37.7, 40.6, 46.6
1.5	Ten concentrations: 4.7, 9.7, 14.7, 20.1, 25.4, 30.7, 36, 41.4, 45.9, 50.1	Ten concentrations: 2.3, 4.8, 7.6, 10.6, 13.9, 17.3, 21.1, 25, 28.6, 32.1
2.5	Nine concentrations: 5.6, 10.6, 15.6, 21.1, 26, 31, 35.4, 40.4, 45.4	Nine concentrations: 2.8, 5.3, 8.1, 11.3, 14.3, 17.6, 20.7, 24.4, 28.3
3.5	Five concentrations: 5.1, 10.1, 15, 20.1, 25.1	Five concentrations: 2.5, 5.1, 7.8, 10.7, 13.8

## Data Availability

The raw data supporting the conclusions of this article will be made available by the authors on request.
